# Antiviral activity of lipoxygenase against severe fever with thrombocytopenia syndrome virus

**DOI:** 10.1093/procel/pwae061

**Published:** 2024-10-29

**Authors:** Shuang Li, Xiaojie Zheng, Yunfa Zhang, Lingyu Zhang, Tong Yang, Hao Li, Caiyu Zhou, Xiao-Ai Zhang, Li-Zeng Gao, Wei Liu

**Affiliations:** Department of Infectious Disease Epidemiology, State Key Laboratory of Pathogen and Biosecurity, Academy of Military Medical Science, Beijing 100071, China; Department of Infectious Disease Epidemiology, State Key Laboratory of Pathogen and Biosecurity, Academy of Military Medical Science, Beijing 100071, China; Department of Infectious Disease Epidemiology, State Key Laboratory of Pathogen and Biosecurity, Academy of Military Medical Science, Beijing 100071, China; Department of Infectious Disease Epidemiology, State Key Laboratory of Pathogen and Biosecurity, Academy of Military Medical Science, Beijing 100071, China; Department of Infectious Disease Epidemiology, State Key Laboratory of Pathogen and Biosecurity, Academy of Military Medical Science, Beijing 100071, China; Department of Research and Development, Luoyang TMAXTREE Biotechnology Co., Ltd., Luoyang 471000, China; Department of Infectious Disease Epidemiology, State Key Laboratory of Pathogen and Biosecurity, Academy of Military Medical Science, Beijing 100071, China; Department of Epidemiology and Health Statistics, School of Public Health, Anhui Medical University, Hefei 230032, China; CAS Engineering Laboratory for Nanozyme, Key Laboratory of Biomacromolecules (CAS), Institute of Biophysics, Chinese Academy of Sciences, Beijing 100101, China; Department of Infectious Disease Epidemiology, State Key Laboratory of Pathogen and Biosecurity, Academy of Military Medical Science, Beijing 100071, China; CAS Engineering Laboratory for Nanozyme, Key Laboratory of Biomacromolecules (CAS), Institute of Biophysics, Chinese Academy of Sciences, Beijing 100101, China; Department of Infectious Disease Epidemiology, State Key Laboratory of Pathogen and Biosecurity, Academy of Military Medical Science, Beijing 100071, China; Department of Epidemiology and Health Statistics, School of Public Health, Anhui Medical University, Hefei 230032, China

**Dear Editor,**

Severe fever with thrombocytopenia syndrome (SFTS) is an emerging tick-borne infectious disease caused by Dabie bandavirus, also known as SFTS virus (SFTSV), a novel *phlebovirus* in the family *Phenuiviridae* of the order *Bunyavirales.* The disease was first identified in China and subsequently reported in South Korea and Japan, leading to a high fatality rate of 12%–50% (Yu et al., 2011). The disease causes a wide clinical spectrum, ranging from mild febrile disease accompanied by thrombocytopenia and/or leukocytopenia, to hemorrhagic fever, clinical encephalitis, multiple organ failure (MODS), sepsis, and disseminated intravascular coagulation, and even death (Liu et al., 2014). Despite the high case fatality, vaccines, or specific antivirals for SFTSV are currently not available. A recent study has demonstrated a clinical benefit of favipiravir in reducing viral loads and increasing survival rates in human patients, which, however, are effective only when administered at early disease and to a portion of patients (Li et al., 2021). There is an urgent requirement for the development of effective anti-SFTSV drugs and therapeutic strategies may be perceived as critical.

Lipoxygenases (LOXs) are enzymes that oxidize polyunsaturated fatty acids, leading to lipid peroxidation. LOXs are found in various organisms, including humans, animals, plants, fungi, and bacteria (Chrisnasari et al., 2022). In humans, there are six known isoforms of LOX: 15-LOX, 15-LOX-2, 12-LOX, 12R-LOX, eLOX-3, and 5-LOX (Mashima and Okuyama, 2015). LOXs and their metabolites play important roles as signaling molecules involved in biological processes such as cell proliferation and carcinogenesis, inflammation, and metabolic disorders (Kuhn et al., 2015). Recent studies have demonstrated the association between lipoxygenase and viral infection. For instance, the enzyme responsible for producing lipid mediator protectin D1 (PD1), which attenuates influenza virus replication through RNA export machinery is identified as 12/15-LOX (Morita et al., 2013). In addition, Daidzein activates anti-influenza activity by suppressing intracellular replication of influenza virus via mitogen-activated protein kinase kinase (MEK) /extracellular signal-regulated kinase (ERK) pathway by activating 5-LOX (Horio et al., 2023). In addition to this, lipid-protein assemblies like biomembranes can be modified structurally or functionally by oxidation from LOXs (Kuhn et al., 2015). A previous report has reported that iron oxide nanozyme disrupts the viral envelope and diminishes H1N1 virus infection through triggering lipid peroxidation (Qin et al., 2019). However, it remains unknown whether LOX oxidizes viral envelopes to prevent viral infection. Here, we demonstrated that soybean LOX, which shares both amino acid sequence similarity and biochemical properties with human 15-LOX enzymes (Prigge et al., 1996), effectively inhibits the entire infection process of SFTSV *in vitro*.

To determine the potential antiviral function of LOX, we initially assessed its cytotoxicity on Huh 7 cells. The results showed that concentrations of LOX ranging from 0 to 8 mg/mL had no detrimental effect on cell survival (Fig. 1A). Subsequently, we evaluated the antiviral effect of LOX on SFTSV in Huh7 cells by examining intracellular and supernatant SFTSV vRNA at 24 hpi. Our findings demonstrated a dose-dependent inhibitory effect of LOX treatment on the entire infection process of SFTSV (entire stage, 2 h pre-infection to 24 hpi) (Fig. 1B–D). Similar inhibitory effects were observed in terms of viral nucleoprotein production under LOX treatment as confirmed by Western blot assay and immunofluorescence assay (IFA) (Fig. 1E and 1F). Furthermore, we validated the inhibitory activity of LOX on SFTSV infection in THP-1 cells and HUVECs, which are susceptible to SFTSV infection (Sun et al., 2014; Zhang et al., 2023). Consistent with the result obtained from Huh7 cells, dose-dependent anti-SFTSV effects were observed for LOX treatment (Fig. S1).

**Figure 1.**
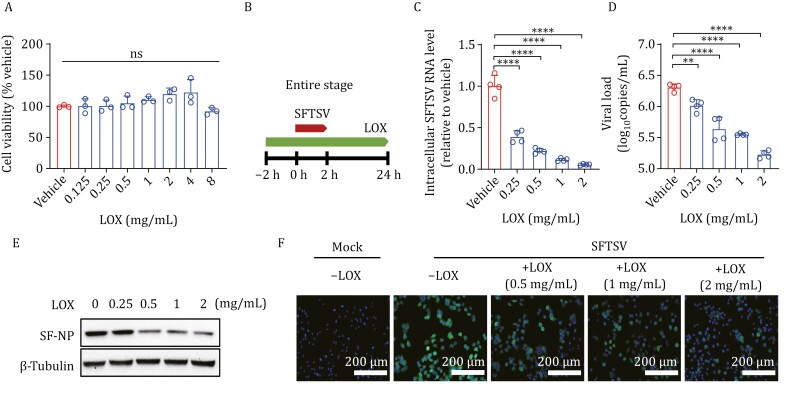
**The cytotoxicity and anti-SFTSV activity of LOX.** (A) Huh7 cells were treated with indicated concentrations of LOX for 24 h. Cell viability was evaluated using CCK-8 assays; *n* = 3. (B) A schematic diagram illustrating the entire process of LOX treatment. (C–F) Huh7 cells infected with SFTSV (MOI = 1) were exposed to indicated concentrations of LOX from 2 h before infection until 24 hpi. Intracellular SFTSV RNA levels (C) and supernatant viral copies (D) were quantified using RT-qPCR; *n* = 4, while NP levels were measured by Western blot (E). Microscopy of Huh7 cells immunostained for SFTSV NP, and DAPI at 24 hpi (F). Scale bars: 200 μm. Data shown are means ± SD. The two-sided *P* values were examined using one-way ANOVA followed by Tukey’s multiple comparisons test for comparison of continuous variables among multiple groups. *P < 0.05; **P < 0.01; ***P < 0.001; and ****P < 0.0001.

To investigate whether LOX possesses a virucidal effect against SFTSV virions, we incubated varying concentrations of LOX with SFTSV for two hours at 37°C and then assessed the infectivity levels of treated viruses (Fig. S2A). As shown in Fig. S2B, both intracellular and supernatant vRNA levels were reduced. In addition, western blot assay revealed a decrease in the expression level of SFTSV nucleoprotein when the virus was pre-incubated with LOX before adding it to the cell culture medium (Fig. S2C). Subsequently, time-of-addition analysis was conducted to identify specific stages during SFTSV replication that were inhibited by LOX. We added LOX during both the entry process (0–2 hpi) and post-entry process (2–24 hpi), followed by harvesting infected cells at 24 hpi (Fig. S2A). The levels of intracellular and supernatant vRNA were quantified using RT-qPCR. A corresponding inhibitory effect was observed at both the virus entry and post-entry phases, with intracellular vRNA levels being reduced significantly (Fig. S2D–G). Consistently, treatment with LOX also resulted in a decrease in viral nucleoprotein present in cells during both phases (Fig. S2H and S2I) These results collectively suggest that besides destabilizing virus particles, LOX also interferes with virus entry as well as subsequent stages after entry; effectively inhibiting SFTSV infection.

To validate the virucidal effect of LOX on SFTSV virions, we used immunofluorescence analysis to observe the infectivity of SFTSV pre-incubated with LOX in Huh 7 cells. Immunofluorescence assay demonstrated a significant dose-dependent suppression of SFTSV infectivity by LOX (Fig. 2A). LOX, acting as a lipid-peroxidizing enzyme, can modify the structure and function of complex ester lipid or lipid-protein assemblies (Kuhn et al., 2015). To test whether LOX alters the stability of virions by oxidizing the virus lipid envelope, we initially measured maleic dialdehyde (MDA), a biomarker for lipid peroxidation, produced through incubating LOX with SFTSV. As shown in Fig. 2B, both increasing concentration of LOX and culture duration of purified SFTSV correlated positively with a gradual increase in MDA. Furthermore, pretreatment with LOX revealed substantial damage to the viral lipid envelope, altering its morphology (Fig. 2C). The Gn and Gc glycoproteins (GP) form heterodimers on the SFTSV envelope to facilitate virus-cell binding. Western blot assay indicated that GP was degraded when exposed to LOX, while NP protein remained unaffected (Fig. 2D). These observations suggest that treatment with LOX may disrupt the integrity of the viral membrane of SFTSV, potentially compromising its ability to efficiently enter host cells.

**Figure 2.**
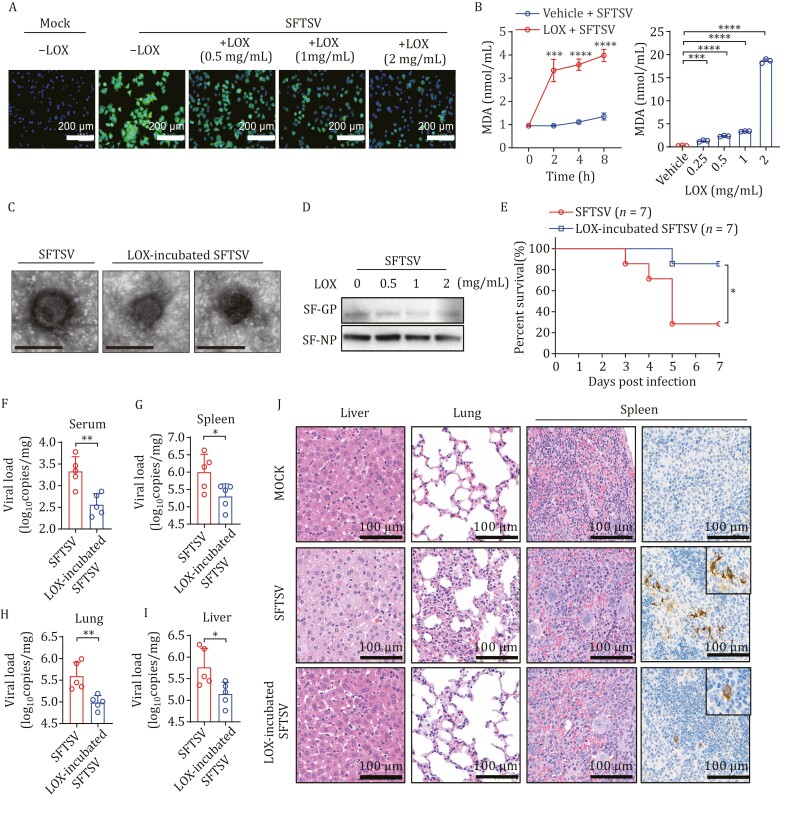
**Lipoxygenase disrupts the virion stability of SFTSV.** (A) Huh 7 cells were infected with SFTSV that had been pre-incubated with LOX or vehicle for 24 h, and then fixed to detect viral replication by immunofluorescence staining of SFTSV NP protein and DAP. Scale bars: 200 μm. (B) The level of lipid peroxidation (MDA detection) was measured after incubating with LOX (1 mg/mL) for 0–8 h or different concentrations of LOX for 2 h; *n* = 3. (C) TEM image of SFTSV pre-incubated with LOX or vehicle. Scale bars, 100 nm. (D) Western blot analysis of GP protein, and NP of SFTSV incubated with LOX. (E) Survival curves of mouse model intraperitoneally injected with SFTSV pre-incubated with LOX (2 mg/mL) or vehicle (*n* = 7 per group). (F–I) Viral copies in serum, spleen, lung, and liver from mice injected with SFTSV pre-incubated with LOX or vehicle were measured using RT-qPCR at 5 dpi (*n* = 5 per group). (J) The H&E assays and immunohistochemistry staining revealed pathological lesions in the liver, lung, and spleen sections collected at 5 dpi from mice treated with SFTSV pre-incubated with LOX or vehicle. Data shown are means ± SD. The two-sided *P* values were examined using Student’s *t* test for comparison of variables between two groups (B and F–I) or one-way ANOVA followed by Tukey’s multiple comparisons test for comparison of continuous variables among multiple groups (B). The log-rank (Mantel-Cox) test was used to analyze time-to-event data (E). *P < 0.05; **P < 0.01; ***P < 0.001; ****P < 0.0001; ns, no significance.

The binding assay confirmed that LOX interferes with virus entry, evidenced by the significant reduction in SFTSV vRNA levels when pre-incubated with LOX compared to vehicle (Fig. S3A). Next, the internalization assay showed that no difference in intracellular vRNA between LOX-treated and vehicle-treated samples (Fig. S3B). These results indicate that LOX affects the binding of SFTSV to cellular receptors rather than SFTSV internalization. Furthermore, when pre-incubated with other enveloped virus, including H1N1 influenza virus (family *Orthomyxoviridae*), vesicular stomatitis virus (VSV, family *Rhabdoviridae*), and herpes simplex virus encephalitis HSV-1 (family *Herpesviridae*), LOX significantly reduced viral infection (Fig. S4). Conversely, enterovirus 71, EV71 (family *Picornaviridae*), which lacks envelope structure, maintained effective infection in Huh 7 cells under conditions where it was pre-incubated with LOX (Fig. S4). These findings suggest that LOX may possess broad-spectrum anti-viral activity against enveloped viruses.

The virucidal effect of LOX was further evaluated in a lethal mouse model, where six-week-old C57BL/6J mice were pretreated with anti-IFNAR1-blocking antibodies to assess the pathogenicity of SFTSV (Li et al., 2019). Compared to mice infected with intact SFTSV, fatality rate, and viral loads in serum, spleen, lung, and liver tissues from mice infected with LOX pre-incubated SFTSV were significantly lower (Fig. 2E–I). Moreover, hematoxylin and eosin (H&E) staining revealed that ballooning degeneration induced by SFTSV in hepatocytes was attenuated in mice infected with LOX pre-incubated virus. Severe pneumonia characterized by inflammatory cellular infiltration along with alveolar wall edema and thickening was observed in lung tissues; an increased number of megakaryocytes present in spleen tissues from mice infected with intact SFTSV also occurred. In comparison, histopathological damage was ameliorated in the group exposed to LOX pre-incubated SFTSV (Fig. 2J). Similar results were also observed through immunohistochemical staining for SFTSV antigen expression in the spleen (Fig. 2J); although viral antigen expression was still detected, the number of cell foci decreased among mice infected with LOX pre-incubated SFTSV. These findings provide evidence that LOX could suppress the replication and spread of viruses by effectively deactivating them.

The therapeutic efficacy of LOX on SFTSV infection was further evaluated. The mice were divided into two groups and received either a vehicle (PBS) or a 100 mg/kg/day dose of LOX. Compared to the vehicle-treated group, LOX treatment resulted in reduced fatality rate and decreased viral loads in serum and tissue samples including spleen, lung, and liver (Fig. S5). Collectively, these results indicate that LOX effectively mitigates lethality in mice while enhancing their resistance to infection.

In our study, we identified LOX as a potent inhibitor of SFTSV infection *in vitro*. LOX functions by catalyzing lipid peroxidation of the viral lipid envelope and thus destabilize SFTSV virions *in vitro*. Furthermore, pre-incubation with LOX resulted in reduced mortality and alleviated tissue lesions in a mouse model infected with SFTSV. Interestingly, treatment with LOX also decreased SFTSV infection and increased survival rate in mice. It is worth noting that LOXs possess the capacity to oxygenate polyenoic fatty acids, and the incorporation of a hydrophilic peroxide group into the hydrophobic tail of a fatty acid alters the physico-chemical properties of the ester lipids, thereby impairing membrane function (Kuhn et al., 2015). The viral envelope membrane primarily originates from the host cell membrane (phospholipid layer and membrane proteins) and contains some viral glycoproteins. Qin et al., (2019) demonstrated that IONzymes catalyze lipid peroxidation in the viral lipid envelope, compromising the integrity of the neighboring proteins. Our findings indicate that LOX also exhibits the potential to disrupt the envelope structure of SFTSV, consequently hindering virus-cell binding and reducing viral adsorption onto host cells. The glycoprotein (GP), cleaved into Gn and Gc, plays a crucial role in mediating SFTSV infection within host cells (Yuan et al., 2021). Our Western blot analysis confirmed GP degradation while leaving nucleoprotein unaffected due to LOX-induced lipid peroxidation damaging GP or causing structural changes through envelope disintegration. Importantly, we have demonstrated a broad-spectrum virucidal efficacy of LOX against enveloped viruses *in vitro* which holds potential as a broad-spectrum antiviral agent for other enveloped viral diseases. Further investigation is warranted to elucidate the intricate mechanisms underlying LOX’s inhibitory effects on SFTSV. In summary, we have demonstrated that LOX exerts a virucidal effect against SFTSV, through the peroxidation of the viral lipid envelope. As a result, LOX poses as a promising therapeutic and preventive candidate for SFTSV and potentially other enveloped viruses.

## Supplementary data

Supplementary data is available at *Protein & Cell* online at https://doi.org/10.1093/procel/pwae061.

pwae061_suppl_Supplementary_Figures_S1-S5

## Footnotes

The authors would like to thank Prof. Wen-hai Feng from China Agricultural University for kindly providing HSV-1 and VSV. We would also like to thank the administrative personnel of the State Key Laboratory of Pathogen and Biosecurity for their assistance and our colleagues for their comments. The study was supported by the National Natural Science Foundation of China (22121003). All authors declare that no competing interests exist and their agreement to participate and publish. Animal experiment was approved by the Institutional Animal Care and Use Committee (IACUC-IME-2023-003) of the State Key Laboratory of Pathogens and Biosecurity (Beijing, China) and was performed in accordance with the National Institutes of Health guidelines under protocols. All data supporting the results can be found in this paper and its Supplementary Materials. All other relevant data can be obtained from the corresponding authors upon request. All authors reviewed and approved the paper. W.L., L-Z.G., and X.A.Z. developed the research plan and experimental strategy. S.L., X.J.Z., and T.Y. performed the *in vitro* experiments. S.L., Y.F.Z., and L.Y.Z. performed the animal experiments. S.L., and X.J.Z. performed statistical analysis. L-Z.G., H.L., and C.Y.Z. provided expertise in *in vitro* and animal experiments. W.L., L-Z.G., S.L., and X.J.Z. wrote the manuscript.
