# Supplementary material for: Antiviral activity of lipoxygenase against severe fever with thrombocytopenia syndrome virus
Source: Protein Cell. 2024 Oct 29;16(2):143–7. doi: 10.1093/procel/pwae061 (PMC11786719; doi:10.1093/procel/pwae061)
Supplement: pwae061_suppl_Supplementary_Figures_S1-S5 [file pwae061_suppl_supplementary_figures_s1-s5.pdf]

Supplementary Materials

Supplementary Information and Methods

Cells and viruses

Vero, THP-1, HUVEC cells were obtained from the American Type Culture Collection, and Huh 7 cells were obtained from the China Center for Type Culture Collection. Vero, Huh7 and HUVEC cell lines were cultured in Dulbecco's modified Eagle's medium (DMEM; Invitrogen, USA) supplemented with 10% FBS, 10 nM HEPES and 1% antibiotics/antimycotic (Gibco). THP-1 cells were maintained in RPMI 1640 medium (Gibco) containing 10% FBS. All the cells were cultured at 37°C in a humidified atmosphere with 5% CO_2.

The SFTSV strain HBMC16_human_2015 was obtained from the National Virus Resource Centre (Zhang et al., 2023a). Herpes simplex virus encephalitis (HSV-1) and vesicular stomatitis virus (VSV) strains were obtained from China Agricultural University. H1N1 influenza strain was obtained from Engineering Laboratory for Nanozyme, Institute of Biophysics, Chinese Academy of Sciences. All viruses were propagated in Vero cells and experiments involving these viruses were performed within biosafety level 2 (BSL-2) or level 3 (BSL-3) facilities, following the institutional biosafety operating procedures.

Antibody and reagent

The following antibodies were purchased from the indicated manufacturers: LC3B Rabbit mAb (#3868), Phospho-AMPKα (Thr172) Rabbit mAb (#2535), AMPKα antibody (#2532), phospho-mTOR (Ser2448) Rabbit mAb (#5536) and mTOR Rabbit

mAb (#2983) were purchased from Cell Signaling Technology (CST; USA). Anti-β-Tubulin Mouse Monoclonal Antibody (#C1340) was purchased from Applygen Technologies. HRP-conjugated goat anti-Mouse IgG (#SA00001-1) and HRP-conjugated goat anti-Rabbit IgG (#SA00001-2) were purchased from Proteintech. SFTSV NP antibody and GP antibody were prepared and preserved by our laboratory (Li et al., 2019). Soybean LOX lyophilized powder (#L7395) was purchased from Sigma-Aldrich.

Cytotoxicity assay

Huh 7 cells were seeded in a 96-well plate were treated with different concentrations of LOX (Sigma, Germany) for 24 hrs. Cell viability was measured by Cell Counting kit-8 (CCK-8; Beyotime, #c0038) following the supplier's guidelines.

Evaluation of LOX inhibition effects the SFTSV infection process

The inhibitory effect of LOX on the SFTSV infection (MOI = 1) was assessed using infection assays involving different levels of drug treatment (0.25, 0.5, 1, and 2 mg/mL) or vehicle at different stages of infection, as described in previous studies (Li et al., 2021b; Shen et al., 2022; Zhang et al., 2023b). For entire stage assay, Huh7 cells were treated with LOX from 2 h pre-infection to 24 h post infection, as well as infected with SFTSV (0 h) and the initial inoculum was removed at 2 h post infection. HUVEC and THP-1 cells were treated with LOX and SFTSV as described above. For virion stability assay, SFTSV was premixed with each dilution of LOX and incubated at 37°C for 2 h, following which the mixture was added to the Huh7 cells and maintained at 37°C for 2 h. Next, cells were washed thrice using phosphate buffer saline (PBS), and a fresh

medium was added. For the virus entry assay, Huh7 cells were incubated with a mixture containing both SFTSV and LOX at 37°C for 2 h. The cells were washed thrice using PBS, and a fresh medium was added. For the virus post-entry assay, infect Huh 7 cells with SFTSV at 37°C for 2 h, then rinse twice with PBS to remove free virus particles. Then, fresh medium with different concentrations of LOX was added to the cells. Infected cells and supernatant were collected at 24 h post infection (hpi) and subjected to RT-qPCR and western blot.

Virus binding assay

After pretreatment with LOX or vehicle for 2 h at 37°C, Huh7 cells were incubated at 4°C for 15 min. Processed SFTSV was subsequently added, and the cells were incubated with a mixture containing virus and LOX or vehicle at 4°C for 2 h. Finally, the cells were harvested after being washed twice with cold PBS, and analyzed using RT-qPCR.

Virus internalization assay

Huh7 cells were incubated with SFTSV at MOI of 1 for 2 h at 4°C. Subsequently, the virus was removed and the cells were washed twice with cold PBS. Ammonium chloride (NH_4Cl) was then added to inhibit fusion, followed by the addition of fresh medium containing varying concentrations of LOX. The cell cultures were changed to·37°C. and following culture for 3 h, cells were washed with PBS and treated with trypsin to eliminate non-internalized virions. The relative level of internalized virions was determined using RT-qPCR (Li et al., 2019).

RT-qPCR assay

Total RNA was extracted from cells using the RNAprep pure Cell Kit (TIANGEN, #DP430), and from cell culture supernatant using the TIANamp Virus RNA Kit (TIANGEN, #DP315-R), following the manufacturer's instructions. Real-time reverse-transcription PCR with SYBR Green was used for relative quantitation of intracellular virus RNA (vRNA), while Taq Man was used for absolute quantitation of cell culture supernatant virus RNA (vRNA). The real-time PCR primers and probe sequence were shown as below:

SFTSV Forward: 5'-AGCCTAATTGGATATGTCAAATTGC-3'

SFTSV Reverse: 5'-CGGGTGAAGTGGCTGAAGG-3'

SFTSV probe: 5'-FAM-AGCAGCAGCAGCAACCTCAGCAGC- BHQ1-3'

GAPDH-human Forward: 5'-GAAGGTGAAGGTCGGAGTC-3'

GAPDH-human Reverse: 5'-GAAGATGGTGATGGGATTTC-3'

SFTSV Forward: 5'-CTCACTCATGCCCTCAACGA-3'

SFTSV Reverse: 5'-GATGAACTCACCAGCCCTGC-3'

EV71 Forward: 5'-GCAGCCCAAAAGAACTTCAC-3'

EV71 Reverse: 5'-ATTTCAGCAGCTTGGAGTGC-3'

VSV Forward: 5'-TCGGGAAAGCAGGGGATACA-3'

VSV Reverse: 5'-CCCATCCATGAGCTTTTTCTGTA-3'

HSV-1 Forward: 5'-CCGGCCATCAAGAAGTACGA-3'

HSV-1 Reverse: 5'-CTGGGCTAGCGTGTTGTTCCG-3'

Western blot analysis

The cells were treated as indicated followed by using a lysis buffer. Subsequently, the

lysates were subjected to SDS-polyacrylamide gel electrophoresis with a gradient of 12%–15% ((SurePAGE™, #M00665). The separated proteins were then transferred onto polyvinylidene difluoride (PVDF) membranes (Millipore, # IPVH00010). After blocking with 5% BSA in Tris-buffered saline-Tween 20 (TBST, Solarbio), the membrane was probed with primary antibodies and subsequently incubated with HRP-conjugated secondary antibodies. Protein bands were visualized using an enhanced chemiluminescence kit (Thermo, #34580) and detected using a Fully Automated Chemiluminescence Image Analysis System.

Immunofluorescence assay

Huh 7 cells were infected with SFTSV and incubated with LOX for 2 h. At 24 hpi, the cells were fixed with PBS containing 4% paraformaldehyde for 20 min, washed three times with PBS containing 0.01% Tween-20, and then blocked with a solution of 5% BSA and 0.1% Triton X-100 in PBS for 1.5 h. Subsequently, the cells were incubated overnight at 4 °C with a mouse monoclonal antibody against SFTSV NP, followed by incubation with fluorescein isothiocyanate (FITC) conjugated goat anti-mouse IgG (Transgene, #HS211). Finally, after being washed with PBS, the cells were observed under Leica fluorescence microscopy.

Negative staining Transmission Electron Microscope (TEM) analysis

The cell supernatant was collected from the culture flask by centrifugation at 12000 ×g for 5 min. Subsequently, the clarified supernatant was concentrated using a 50 kDa ultrafiltration tube (Millipore, #UFC9050) to obtain the purified virus solution. The purified SFTSV virus particles were then incubated with PBS or varying doses of LOX

at 37 °C for 2 hours. Following primary fixation with 2.5% glutaraldehyde at 4 °C, negative staining of the virus particles was performed and visualized using TEM (Hitachi High-Tech, HT7700).

Determination of lipid peroxidation

LOX was mixed with SFTSV and incubate at 37 °C, followed by measurement of lipid peroxidation levels using a commercially available MDA assay kit (Nanjing Jiancheng Bioengineering Research Institute, #A003-4-1) according to the manufacturer's instructions. The concentration of MDA was determined based on the absorbance of TBA reactive substances at 530 nm.

Virucidal effect of LOX on different viruses

For the assessment of LOX's virucidal activity against H1N1, VSV, HSV-1 and EV71 virion stability, all viruses were pre-incubated with various dilutions of LOX at 37°C for 2 hrs. The virus-LOX mixture was then added to the Huh7 cells and incubated at 37 °C for an additional 2 hrs. Subsequently, the medium was replaced with fresh medium and the infected cells were cultured at 37°C for 24 h. Intracellular vRNA was analyzed using RT-qPCR. To assess the infectivity of H1N1, a hemagglutination (HA) assay was performed as previously described (Qin et al., 2019).

Animal study

Six-week-old female C57BL/6J mice were obtained from (Beijing Vital River) and kept in an environmentally controlled pathogen-free animal facility at the State Key Laboratory of Pathogens and Biosecurity (Beijing, China). Animal experiment was approved by the Institutional Animal Care and Use Committee (IACUC-IME-2023-003)

and was performed in accordance with the National Institutes of Health guidelines under protocols. To establish a lethal mouse model for assessing the pathogenicity of SFTSV, pretreatment was performed using anti-interferon alpha/beta receptor subunit 1 (IFNAR1) blocking antibody (BioXCell, #BE0241). C57BL/6 mice received intraperitoneal injection of anti-IFNAR1 IgG (300 μg per mouse) one day prior to infection (Li et al., 2019).

To investigate the virucidal effect of LOX on SFTSV virions, the mice were randomly divided into two groups: SFTSV group and LOX-incubated SFTSV group. On day 0, mice were intraperitoneally injected with 100 μL of virus solution (2×10^5 FFU/mL) that had been incubated with LOX (2 mg/mL) or vehicle for 2 h at 37°C.

For studying LOX inhibition in SFTSV infection, the mice were randomly divided into two groups: SFTSV + vehicle group, and SFTSV + LOX group. Infection experiments involved intraperitoneal inoculation of mice with either 100 μL of virus solution (2×10^5 FFU/mL). Post-infection, LOX or an equivalent volume of vehicle was administered to mice via intraperitoneal injection at a dose of 100 mg/kg/day for a duration of 7 days.

The survival rate of the mice was monitored daily. Following dissection of C57BL/6J mice at 5 dpi, serum samples were collected to detect virus titers, while liver, lung, and spleen samples were collected for examination of virus titers and histopathology (H＆E staining and immunohistochemistry). Immunohistochemistry with a mouse monoclonal antibody against SFTSV NP.

Statistical analysis

All experiments were conducted in triplicate to ensure reproducibility. Data analysis was performed using GraphPad Prism 7 software (San Diego, CA) and the results were presented as means ± SD. Statistical significance was determined using Student's t-test between the two groups or one-way analysis of variance (ANOVA) followed by Tukey’s multiple comparisons test among multiple groups. The Log-rank (Mantel-Cox) test was used to analyze time-to-event data. Significance levels were denoted as *P < 0.05; **P <0.01; ***P < 0.001; and ****P < 0.0001; ns, no significance.

Supplementary Figures

Supplementary Figure 1

Figure S1. The anti-SFTSV activity of LOX in THP-1 cells and HUVECs.

(A - D) THP-1 cells (A-B) and HUVECs (C and D) infected with SFTSV (MOI = 1) were exposed to indicated concentrations of LOX from 2 h before infection until 24 hpi. Intracellular SFTSV RNA levels and supernatant viral copies were quantified using RT-qPCR.

Data shown are means ± SD. The two-sided p values were examined using One-way ANOVA followed by Tukey's multiple comparisons test for comparison of continuous variables among multiple groups. *P < 0.05; **P <0.01; ***P < 0.001; ****P < 0.0001; ns, no significance.

Supplementary Figure 2

Figure S2. Lipoxygenase suppresses SFTSV infection during entire lifecycle of SFTSV.

(A) Schematic diagram that determined the antiviral activity interfering with the virion stability, virus entry, and post-entry stage of SFTSV infection in Huh 7 cells.

(B and C) Huh7 cells were treated with SFTSV pre-incubated with LOX or vehicle and the levels of intracellular SFTSV RNA and supernatant viral copies were measured with RT-qPCR (B), while NP levels were measured by western blot (C).

(D - I) Huh7 cells infected with SFTSV (MOI = 1) for 2 h were treated with indicated concentrations of LOX for entry or post-entry process. At·24 hpi, the levels of intracellular SFTSV RNA levels (D and E), supernatant viral copies (F and G) were measured with RT-qPCR, and NP levels (H and I) were measured by western blot.

Data shown are means ± SD. The two-sided p values were examined using One-way ANOVA followed by Tukey's multiple comparisons test for comparison of continuous variables among multiple groups. *$P < 0.05$; **$P < 0.01$; ***$P < 0.001$; ****$P < 0.0001$; ns, no significance.

Supplementary Figure 3

Figure S3. The effect of LOX-treated SFTSV on binding and internalization.

(A) The binding assay was performed to determine the effect of LOX-treated SFTSV binding to Huh 7 cells (up). The vRNA of bound SFTSV virions was measured using RT-qPCR (down).

(B) The internalization assay under a temperature shift was carried out to determine the effect of LOX on the internalization of SFTSV in Huh7 cells (up). Intracellular levels of SFTSV RNA were measured using RT-qPCR (down).

Data shown are means ± SD. The two-sided p values were examined using One-way ANOVA followed by Tukey's multiple comparisons test for comparison of continuous variables among multiple groups. *P < 0.05; **P <0.01; ***P < 0.001; ****P < 0.0001; ns, no significance.

Supplementary Figure 4

Figure S4. The broad-spectrum virucidal effect of LOX.

HSV-1, VSV, H1N1, and EV71 were pre-incubated with various dilutions of LOX and then added to the Huh7 cells. At 24 hpi, intracellular vRNA was analyzed using RT-qPCR (A, B, and D), Hemagglutination (HA) assay was performed to assess the infectivity of H1N1 (C).

Data shown are means ± SD. The two-sided p values were examined using One-way ANOVA followed by Tukey's multiple comparisons test for comparison of continuous variables among multiple groups. *P < 0.05; **P <0.01; ***P < 0.001; ****P < 0.0001; ns, no significance.

Supplementary Figure 5

Figure S5. The therapeutic efficacy of LOX on SFTSV infection.

(A) Survival curves of LOX- (n = 7) or vehicle-treated (n = 7) C57BL/6J mice that were pretreated with anti-IFNAR1 IgG antibody and infected with SFTSV (2×10^4 PFU per mouse).

(B - E) Viral copies in serum, spleen, lung, and liver from SFTSV-infected mice with LOX or vehicle treatment were measured using RT-qPCR at 5 dpi (n = 5 per group).

Data shown are means ± SD. The two-sided p values were examined using Student's t test for comparison of variables between two groups (B - E). The Log-rank (Mantel-Cox) test was used to analyze time-to-event data (A). *P < 0.05; **P <0.01; ***P < 0.001; ****P < 0.0001; ns, no significance.

SI References

Li, H., L. K. Zhang, S. F. Li, S. F. Zhang, W. W. Wan, Y. L. Zhang, Q. L. Xin, K. Dai, Y. Y. Hu, Z. B. Wang, X. T. Zhu, Y. J. Fang, N. Cui, P. H. Zhang, C. Yuan, Q. B. Lu, J. Y. Bai, F. Deng, G. F. Xiao, W. Liu and K. Peng 2019a. Calcium channel blockers reduce severe fever with thrombocytopenia syndrome virus (SFTSV) related fatality. Cell Res 29: 739-753. doi: 10.1038/s41422-019-0214-z

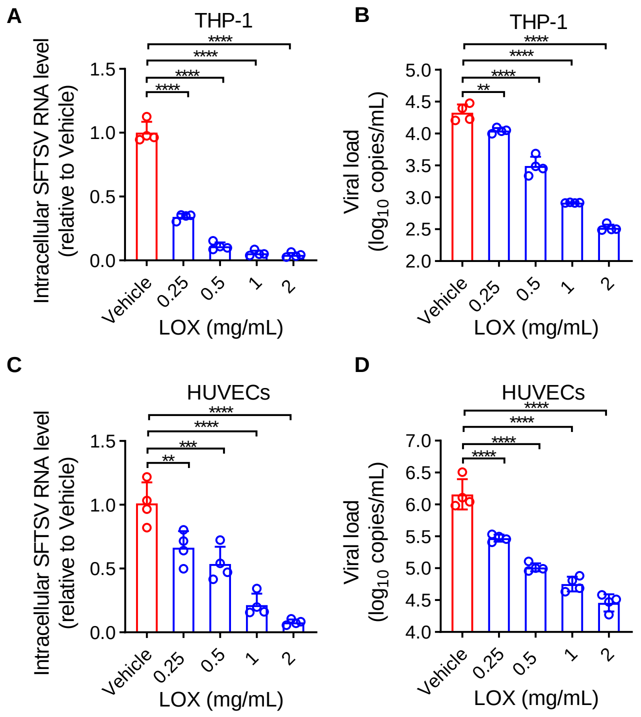

A
THP-1
Intracellular SFTSV RNA level
(relative to Vehicle)
1.5
1.0
0.5
0.0
****
****
****
****
Vehicle
0.25
0.5
1
2
LOX (mg/mL)
B
THP-1
Viral load
(log10 copies/mL)
5.0
4.5
4.0
3.5
3.0
2.5
2.0
****
****
****
**
Vehicle
0.25
0.5
1
2
LOX (mg/mL)
C
HUVECs
Intracellular SFTSV RNA level
(relative to Vehicle)
1.5
1.0
0.5
0.0
****
****
***
**
Vehicle
0.25
0.5
1
2
LOX (mg/mL)
D
HUVECs
Viral load
(log10 copies/mL)
7.0
6.5
6.0
5.5
5.0
4.5
4.0
****
****
****
****
Vehicle
0.25
0.5
1
2
LOX (mg/mL)

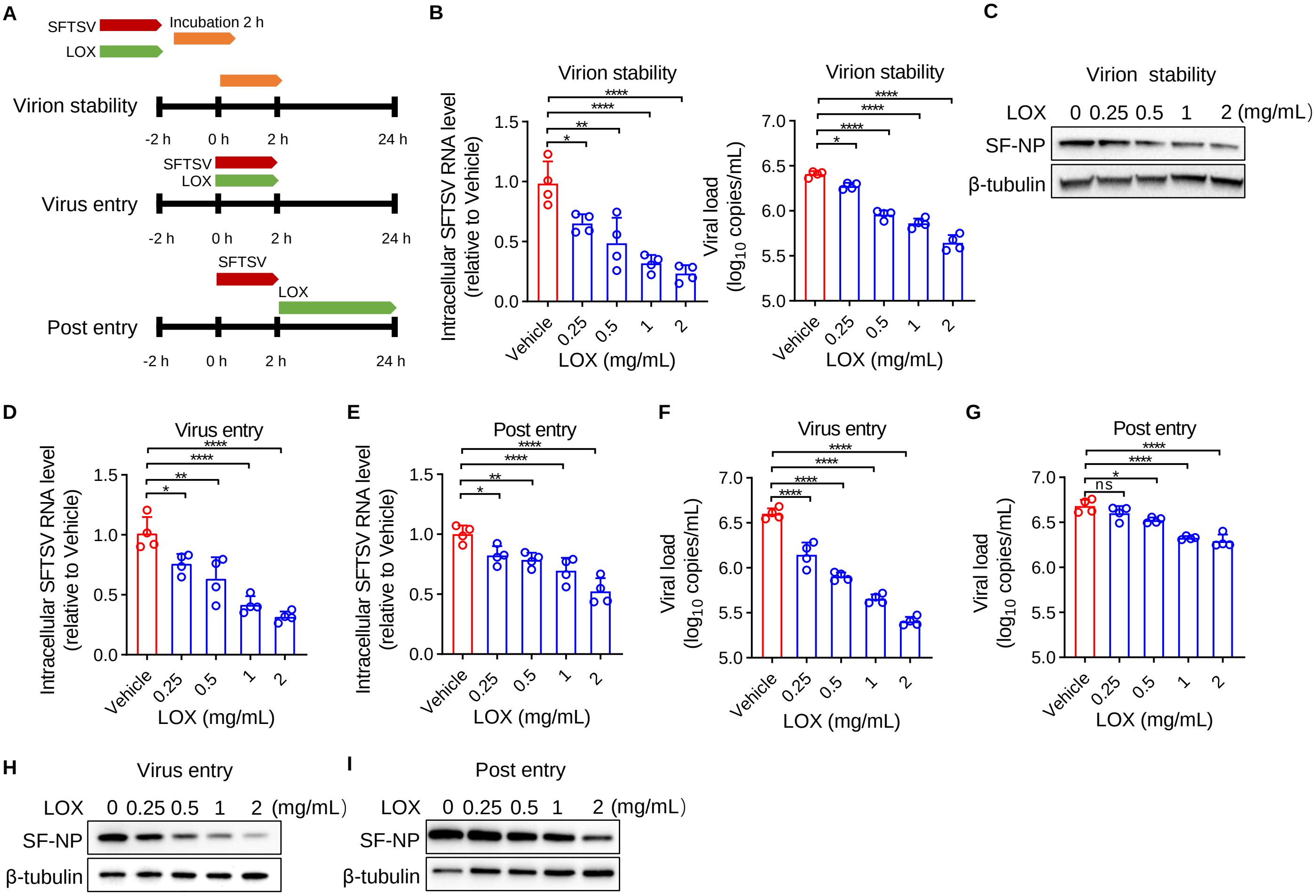

A
SFTSV
LOX
Incubation 2 h
Virion stability
-2 h
0 h
2 h
24 h
Virus entry
Post entry
B
Virion stability
Intracellular SFTSV RNA level (relative to Vehicle)
Viral load (log_{10} copies/mL)
Vehicle
0.25
0.5
1
2
LOX (mg/mL)
C
LOX 0 0.25 0.5 1 2 (mg/mL)
SF-NP
β-tubulin
D
Virus entry
E
Post entry
F
Virus entry
G
Post entry
ns
H
Virus entry
I
Post entry

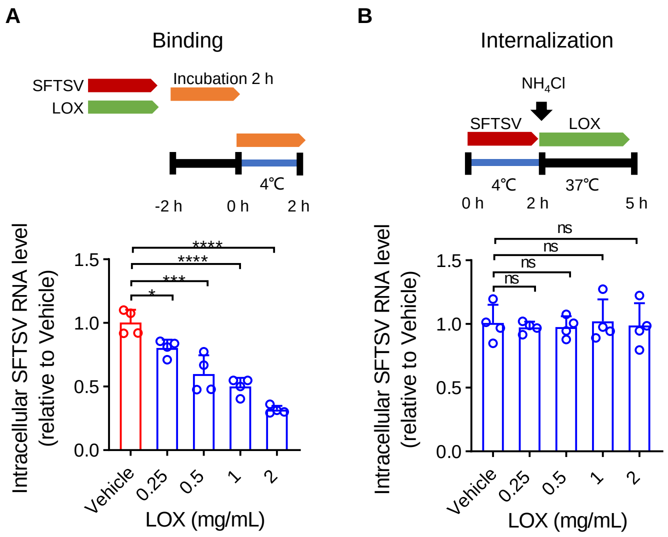
A
Binding
SFTSV
LOX
Incubation 2 h
4℃
-2 h
0 h
2 h
Intracellular SFTSV RNA level (relative to Vehicle)
LOX (mg/mL)
Vehicle
0.25
0.5
1
2
B
Internalization
NH$_4$Cl
SFTSV
LOX
4℃
37℃
0 h
2 h
5 h
ns
Intracellular SFTSV RNA level (relative to Vehicle)
LOX (mg/mL)

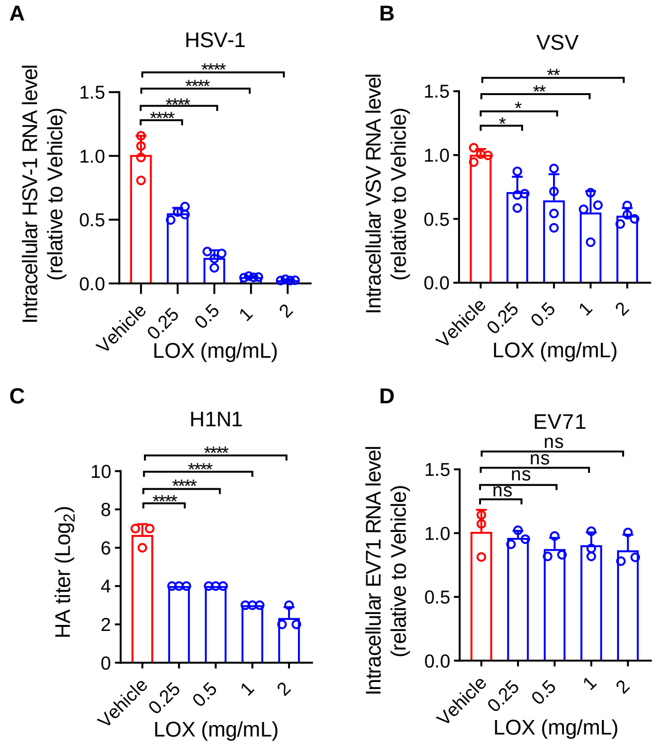

A
HSV-1
Intracellular HSV-1 RNA level (relative to Vehicle)
****
0.0
0.5
1.0
1.5
Vehicle
0.25
0.5
1
2
LOX (mg/mL)
B
VSV
Intracellular VSV RNA level (relative to Vehicle)
**
*
C
H1N1
HA titer (Log2)
0
2
4
6
8
10
D
EV71
Intracellular EV71 RNA level (relative to Vehicle)
ns

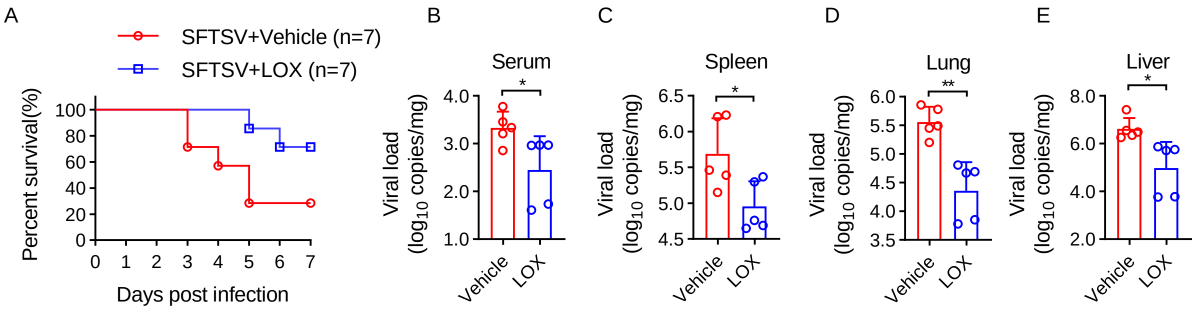

A
SFTSV+Vehicle (n=7)
SFTSV+LOX (n=7)
Percent survival(%)
100
80
60
40
20
0
0 1 2 3 4 5 6 7
Days post infection
B
Serum
*
Viral load (log10 copies/mg)
4.0
3.0
2.0
1.0
Vehicle
LOX
C
Spleen
*
Viral load (log10 copies/mg)
6.5
6.0
5.5
5.0
4.5
Vehicle
LOX
D
Lung
**
Viral load (log10 copies/mg)
6.0
5.5
5.0
4.5
4.0
3.5
Vehicle
LOX
E
Liver
*
Viral load (log10 copies/mg)
8.0
6.0
4.0
2.0
Vehicle
LOX
